# Preclinical evidence for the effective use of TL-895, a highly selective and potent second-generation BTK inhibitor, for the treatment of B-cell malignancies

**DOI:** 10.1038/s41598-023-47735-z

**Published:** 2023-11-21

**Authors:** Samantha M. Goodstal, Jing Lin, Timothy Crandall, Lindsey Crowley, Andrew T. Bender, Albertina Pereira, Maria Soloviev, John S. Wesolowski, Riham Iadevaia, Sven-Eric Schelhorn, Edith Ross, Federica Morandi, Jianguo Ma, Anderson Clark

**Affiliations:** 1 Research Unit Oncology, EMD Serono Research and Development Institute, Inc., 45A Middlesex Turnpike, Billerica, MA 01821 USA; 2 Research Unit Immunology, EMD Serono Research and Development Institute, Inc., Billerica, MA 01821 USA; 3 Protein Engineering Antibody Technologies, EMD Serono Research and Development Institute, Inc., Billerica, MA 01821 USA; 4 Oncology Bioinformatics Quantitative Pharmacology and Drug Disposition (QPD) Biopharma, Merck KGaA, 64293 Darmstadt, Germany; 5 Discovery and Development Technologies, EMD Serono Research and Development Institute, Inc., Billerica, MA 01821 USA

**Keywords:** Haematological cancer, Lymphoma

## Abstract

TL-895 (formerly known as M7583) is a potent, highly selective, adenosine triphosphate (ATP)-competitive, second-generation, irreversible inhibitor of Bruton’s tyrosine kinase (BTK). We characterized its biochemical and cellular effects in in vitro and in vivo models. TL-895 was evaluated preclinically for potency against BTK using IC_50_ concentration–response curves; selectivity using a 270-kinase panel; BTK phosphorylation in Ramos Burkitt’s lymphoma cells by ProteinSimple Wes analysis of one study; anti-proliferative effects in primary chronic lymphocytic leukemia (CLL) blasts; cell viability effects in diffuse large B-cell lymphoma (DLBCL) and mantle-cell lymphoma (MCL) cell lines; effects on antibody-dependent cell-mediated cytotoxicity (ADCC) from Daudi cells and chromium-51 release from human tumor cell lines; and efficacy in vivo using four MCL xenograft model and 21 DLBCL patient-derived xenograft (PDX) models (subtypes: 9 ABC, 11 GCB, 1 Unclassified). TL-895 was active against recombinant BTK (average IC_50_ 1.5 nM) and inhibited only three additional kinases with IC_50_ within tenfold of BTK activity. TL-895 inhibited BTK auto-phosphorylation at the Y223 phosphorylation site (IC_50_ 1–10 nM). TL-895 inhibited the proliferation of primary CLL blasts in vitro and inhibited growth in a subset of activated DLBCL and MCL cell lines. TL-895 inhibited the ADCC mechanism of therapeutic antibodies only at supra-clinical exposure levels. TL-895 significantly inhibited tumor growth in the Mino MCL xenograft model and in 5/21 DLBCL PDX models relative to vehicle controls. These findings demonstrate the potency of TL-895 for BTK and its efficacy in models of B-cell lymphoma despite its refined selectivity.

## Introduction

Ibrutinib is a first-generation, small-molecule inhibitor of Bruton’s tyrosine kinase (BTK). Its approval in patients with CLL and MCL^1,2^ marked the advent of a new era in the treatment of B-cell malignancies, based on clinical efficacy in previously difficult to treat lymphomas, such as treatment-resistant/refractory chronic lymphocytic leukemia (CLL), mantle cell lymphoma (MCL), and Waldenström’s macroglobulinemia^3–6^. Ibrutinib is associated clinically with off-target effects on epidermal growth factor receptor (EGFR), interleukin-2 inducible T-cell kinase (ITK) and Tec family kinases (e.g. rash, platelet dysfunction, and increased risk of bleeding) and treatment resistance^7,8^.

Ideally, a second-generation BTK inhibitor would maintain the potent antitumor effects of ibrutinib but improve overall tolerability and circumvent ibrutinib’s inhibitory effect on antibody-dependent cell-mediated cytotoxicity (ADCC). The second-generation BTK inhibitor acalabrutinib (ACP-196) was first approved for use in MCL on the basis of phase 2 findings that demonstrated high overall response and durable remission rates, despite a limited complete remission rate^9^. In vivo, acalabrutinib is a more potent and selective inhibitor of BTK than ibrutinib^10,11^ and is associated with similar on-target effects^12^, but with fewer adverse effects^9,13^. The most common adverse events seen in the phase 2 MCL study of acalabrutinib were headache (38%), diarrhea (31%), fatigue (27%), and myalgia (38%), with the most frequent grade ≥ 3 adverse events being neutropenia (10%), anemia (9%) and pneumonia (5%)^9^. Acalabrutinib is also approved in patients with CLL or small lymphocytic lymphoma.

TL-895 (formerly known as M7583) is a potent, oral, highly selective, adenosine triphosphate (ATP)-competitive, second-generation, irreversible inhibitor of BTK. It covalently binds to the active site of BTK to produce a high degree of occupancy and inhibitory activity in mice that persists after clearance from circulation^14^. Here we present a comparison of the biochemical and cellular effects of TL-895 with those of ibrutinib and acalabrutinib using in vitro and in vivo models of B-cell malignancies.

## Methods

### Kinase assays

#### Potency

Purified full-length recombinant BTK (Carna Biosciences #08–080) was diluted in buffer (see Supplementary Material) to a final concentration of 0.05 ng/µL with 75 µM ATP and 1 µM of the peptide FITC-AHA-EEPLYWSFPAKKK-NH2 (Tufts Core Facility, Boston, MA, USA) into microtiter plates. TL-895 (0.038–10,000 nM) was added and the samples were incubated at room temperature for 90 min (min) before the addition of stop buffer containing 10 mM ethylenediamine tetra-acetic acid (EDTA; see Supplementary Material). Samples were read on a Caliper LC3000 (Caliper Life Sciences, Waltham, MA, USA) using an Off-Chip mobility shift assay format to measure percentage conversion of substrate to product, from which half maximal inhibitory concentration (IC_50_)–response curves were generated.

#### Selectivity

The Kinase Profiler™ screening panel (EMD Millipore, Burlington, MA, USA) was used to test the inhibitory activity of TL-895 against 270 protein kinases. Kinases were incubated with TL-895 (1 µM) for 20 min to 4 h at room temperature, according to standard kinome screen procedures^13^. The percentage remaining kinase activity (compared with dimethyl sulfoxide control; DMSO) was determined at 1 µM inhibitor. IC_50_ values were determined for the most sensitive kinases (residual kinase activity < 20% of control) and other selected kinases, with concentration-inhibition curves from 30 µM to 1 nM.

### BTK phosphorylation in Ramos cells

Ramos Burkitt's lymphoma cells (American Type Culture Collection [ATCC] Cat# CRL-1596, RRID:CVCL_0597) were maintained in Roswell Park Memorial Institute (RPMI) 1640 medium (Sigma-Aldrich) containing penicillin/streptomycin, 2 mM l-glutamine, and 10% fetal bovine serum, before seeding into 96-well tissue culture plates (8 × 10^7^ cells/mL). Cells were pre-treated with vehicle control (DMSO in RPMI 1640 medium) or TL-895 (final concentrations of 0.001–1 µM) for 30 min at 37 °C and then incubated with anti-human IgM F(ab’)2 antibody (Southern Biotech, Birmingham, AL, USA) at a concentration of 5 µg/mL for 5 min at 37 °C to activate the B-cell receptor (BCR). Cells were collected by centrifugation (500×*g* for 5 min) and, after media aspiration, 150 µL of ice cold Thermo Scientific™ Pierce™ Mammalian Protein Extraction Reagent (M-PER)™ lysis buffer containing Thermo Scientific™ Halt™ protease/phosphatase inhibitor cocktail was added. Finally, cells were resuspended in the lysis buffer and the lysates were frozen to − 80 °C for subsequent measurement of BTK phosphorylation.

BTK phosphorylation was analyzed using the automated Wes instrument (ProteinSimple, San Jose, CA, USA). In brief, 6 µL of lysate was combined with a 1:3000 dilution of primary anti-phospho BTK Y551 (BD Biosciences, Pharmingen, San Diego, CA, USA) or 1:50 anti-phospho BTK Y223 (Cell Signaling Technology) and analyzed in two separate runs of samples from the study according to manufacturer’s instructions. Phosphorylated (p)BTK and GAPDH band intensities were quantified using Compass software (v2.6.6; ProteinSimple; RRID:SCR_015874) and the inhibitory effects were calculated relative to cells activated with anti-human IgM F(ab’)2 antibody and DMSO control.

### In vitro cell proliferation studies and cytotoxicity

Cells from primary blast samples from 21 patients with CLL were treated with TL-895, ibrutinib, or acalabrutinib (0.008–25 µM) and assessed for growth inhibition after 4 days using the Mosaic Spot Assay™ (Mosaic Laboratories). A cytotoxic dose of cisplatin acted as a positive control, with no drug treatment as a negative control.

The potency of TL-895 effects on cell viability was further examined in vitro in a panel of diffuse large B-cell lymphoma (DLBCL) and MCL cell lines, comprising ten germinal B-cell like subtype (GCB)-DLBCL cell lines, six activated B-cell like (ABC)-DLBCL cell lines, and six MCL cell lines (Horizon Discovery Inc, Cambridge MA, USA). Cells were seeded into 384-well plates (500 cells/well) and incubated (37 °C for 24 h) before treatment with varying concentrations of TL-895 for 72 h. ATP levels were analyzed using the ATPlite Luminescence Assay System (Perkin Elmer, UK). Growth inhibition and maximum response were calculated using the Chalice Analyzer (Horizon Discovery Inc).

#### Cytotoxicity

To determine the ability of TL-895 to induce cytotoxicity, the compound was tested in cultures of HepG2 cells (ATCC, Manassas, VA) and primary human hepatocytes (Life Technologies, Carlsbad, CA) by evaluating ATP content. HepG2 cells (human hepatocellular carcinoma [epithelial]) and primary human hepatocytes were cultured in DMEM/F-12 medium supplemented with antibiotics, sodium pyruvate, insulin and 10% fetal calf serum. During the testing steps, the medium was replaced by serum-free media containing bovine serum albumin (BSA) and dexamethasone. All the cultures are incubated at 37 °C in a humidified chamber containing 5% CO_2_. Cultures were deemed suitable for the tests only if the viability (determined by trypan blue staining) was at least 80% (HepG2) and 75% (primary hepatocytes). HepG2 and primary hepatocytes were cultured in 96-well tissue culture plates either for luminescence (2E4/well in 100 µL) or pre-coated with collagen I (3.5E4/well in 100 µL, respectively).

After the appropriate exposure period with TL-895 (24 and 72 h for HepG2, 24 h for primary hepatocytes, doses 50, 10, 5, 1, 0.5, 0.1, 0.05, 0.01 µM), the ATP content was determined using the CellTiter-Glo^®^ kit (Promega, Madison, WI) according to manufacturer’s instructions. TL-895 was tested with 4 replicate wells/dose in 2 independent experiments for each cell type.

### Effects on ADCC

Calcein-AM-loaded Daudi cells (3000 cells/well) from three separate donors were treated with a range of concentrations of TL-895 (0.1–10 µM), ibrutinib, or acalabrutinib (0.3–30 µM) combined with 3 µg/mL rituximab, an ADCC-incompetent version of rituximab, an isotype control antibody, or no antibody, in the presence of primary natural killer cells (15,000 cells/well; Horizon Discovery Ltd, Cambridge, UK) for 4 h in a 384-well plate. ADCC analysis was based on quantitation of Calcein-AM release by luminescence detection on a FLUOstar^®^ Omega (BMG LABTECH).

In a second study, ADCC induced by anti-programmed death-ligand 1 (anti-PD-L1) or anti-EGFR antibody was measured using two cultured human tumor cell lines (A431 and A549, target cells; obtained from ATCC). Target cells were stimulated with human interferon-gamma (200 ng/mL) for 48 h to increase surface expression of PD-L1 target protein. Target cell and effector cells (peripheral blood mononuclear cells) were incubated for 4 h with (a) increasing concentrations of antibody (0.2–1000 ng/mL) and TL-895 (0, 0.05 and 3 µM), or (b) a fixed concentration of antibody (20 ng/mL) and increasing concentrations of TL-895 (8 nM–25 µM). ADCC analysis was based on the quantitation of chromium-51 (^51^Cr) release during lysis of target cells using luminescence detection on a MicroBeta2 2450-0060 liquid scintillation and luminescence counter (PerkinElmer, Waltham, MA, USA).

### Mouse models of B-cell malignancies

All animal studies were conducted in compliance with the guidelines for the care and use of experimental animals and the studies were approved by the Institutional Animal Care and Use Committee (IACUC) of Crown Bioscience (Taicang, China), XenoSTART, EPO-GmbH, Shanghai ChemPartner Co., Ltd and EMD Serono Research and Development Institute following the guidance of the Association for Assessment and Accreditation of Laboratory Animal Care (AAALAC).

#### Dose selection for the in vivo studies

Dose selection of ibrutinib, acalabrutinib, and TL-895 for the MCL mouse model was based on published or in-house preclinical and clinical pharmacokinetic data with the aim of achieving clinically relevant exposure in the animal studies. In patients with MCL, the recommended dose of ibrutinib is 560 mg QD, which produces an area under the curve (AUC) of 956 h·ng/mL with 100% target occupancy^15,16^. In comparison, mice treated orally with ibrutinib at 14.2 mg/kg had a resulting exposure of 569 h·ng/mL^17^. Therefore, to achieve a clinically relevant exposure in mice, an ibrutinib dose of 25 mg/kg daily (QD) by oral gavage (PO) was selected, which would result in an AUC of 1002 h·ng/mL, similar to the level of exposure in patients with MCL.

For acalabrutinib, the current clinically recommended oral dose in MCL is 100 mg twice daily (BID)^18,19^, which results in an exposure of 1111 h·ng/mL^18,19^. There are no published data on the pharmacokinetics of acalabrutinib in mice. The compound was administered at 15 mg/kg BID in mouse xenograft tumor models^11^, giving a total daily dose of 30 mg/kg. This dose was chosen for the current preclinical studies (30 mg/kg QD PO) because it had been tested in the same strain of mice used for these efficacy studies and there were no significant tolerability issues (unpublished observations [J. Ma, J. Lin]).

The TL-895 dose was based on target occupancy and exposure of the compound in mice^14^ and in patients with B-cell malignancies in the phase 1 clinical trial^20^. Similar to the optimum biological dose observed in patients, a dose of 25 mg/kg QD PO in mice resulted in an 8.5-fold greater exposure than that needed for 90–95% target occupancy.

All three compounds were formulated in a solution of 20% kleptose (hydroxypropyl β-cyclodextrin) in 50 mM citrate and administered QD PO during the treatment periods given for each model.

#### Cell-line derived xenograft models of MCL

The activities of TL-895 and ibrutinib were assessed in four cell line-derived xenograft models of MCL: Maver-1, Granta519, Mino, and Jeko-1 (ATCC Cat# CRL-3006, RRID:CVCL_1865).

Maver-1, Granta519, and Mino mouse MCL studies were performed at Shanghai ChemPartner Co., Ltd, Shanghai, China. Five-to-six-week old female severe combined immune deficiency (SCID) mice (Vital River Lab Animal Technology Co., Ltd, Beijing, China) were inoculated with Maver-1, Granta519, or Mino cells (all obtained from ATCC) by subcutaneous (s.c.) injection (0.2 mL containing 5 × 10^7^ cells/mL in a 1:1 suspension of medium:Matrigel). When tumor volumes reached 100–250 mm^3^, mice were assigned to treatment groups (*n* = 6) such that each group had a similar mean tumor volume.

Mice were treated with TL-895, ibrutinib, or vehicle for 14 days (Maver-1 and Granta519 models) or 27 days (Mino model). Tumor volumes were recorded three times per week. Tumor growth inhibition was expressed as the mean percentage treatment (T)/control (C) ratio [%∆T/∆C] and was calculated at the end of treatment.

For mean values > 0:

$${\%}\frac{\Delta {\text{T}}}{\Delta {\text{C}}}=\frac{end\, tumor\, volume\, treatment-start\, tumor\, volume\, treatment}{end\, tumor\, volume\, control-start\, tumor\, volume\, control}\times 100$$

For mean values ≤ 0 (i.e. regression):

$${\%}\frac{\Delta{ \text{T}}}{\Delta {\text{C}}}=\frac{end\, tumor\, volume\, treatment-start \,tumor\, volume\, treatment}{start\, tumor\, volume \,control}\times 100$$

Studies in the Jeko-1 mouse MCL model were performed at EMD Serono Research and Development Institute, Billerica, MA, USA. Six-to-eight-week old female nude mice (Crl:NU-Foxn1nu; Charles River Laboratories, Wilmington) were inoculated with Jeko-1 cells (obtained from ATCC) by s.c. injection (10 × 10^6^ cells in 0.2 mL phosphate buffered saline with Matrigel). Sixteen days after implantation, tumor volumes reached 140–210 mm^3^ and mice were randomized into treatment groups (*n* = 8) such that each group had a similar mean tumor volume. Mice were treated with TL-895, ibrutinib, or vehicle for 20 days. Tumor volumes were recorded twice weekly. Mean %∆T/∆C was calculated at the end of treatment as outlined above.

#### Patient derived xenograft (PDX) models of DLBCL

The antitumor effects of TL-895, ibrutinib, and acalabrutinib were examined in 21 DLBCL PDX models. The research use of patient material in PDX models at Crown Bioscience, XenoSTART and EPO-GmbH was approved by the local Institutional Ethical Review Boards and the patients provided written informed consent. Molecular analysis was performed on samples from each model using the EdgeSeq DLBCL Cell of Origin Assay (HTG Molecular Diagnostics, Tucson, AZ, USA) to confirm ABC-GCB DLBCL subtype.

Stock mice inoculated with human DLBCL cancer tissues were euthanized by progressive hypoxemia with CO_2_ and tumors were harvested. Tumor fragments were used at three different contract research organizations for xenotransplantation into different mouse strains for the following models: (1) 6-week-old female nude (NMRI:nu/nu) or CB17-SCID mice (Javier Labs, France): Ly12638, Ly11212, and Ly13005 (Experimental Pharmacology and Oncology Berlin-Buch GmbH [EPO-GmbH], Germany); (2) 6–8-week-old female BALB/c nude or NOD SCID mice (Beijing HFK Bioscience Co., Ltd. Beijing, China): LY0257, LY2214, LY3604, LY2298, LY2264, LY2318, and LY2345 (Crown Bioscience Inc, Taicang, China); and (3) 6–12-week-old female CB-17 SCID mice (CB17/Icr-Prkdc^scid^/IcrIcoCrl Charles River Laboratories, Wilmington, MA, USA): ST118B, ST949B, ST359, ST949, ST2761B, ST2963, ST1361, ST2078, ST1735B, ST2902, and ST3497 (XenoSTART, San Antonio, TX, USA).

When the average tumor volume was within a pre-determined range (100–300 mm^3^), mice were assigned to treatment groups to balance the mean starting tumor volumes (*n* = 5 for all models except LY13005, which was *n* = 3). Mice were treated with TL-895, ibrutinib, acalabrutinib, or vehicle for 13–25 days, dependent upon the model. Tumor volumes were recorded 2–3 times per week. Mean %∆T/∆C was calculated at the end of treatment as described above for MCL models.

### Bioinformatics

#### Library preparation and sequencing

For each RNA Sequencing (RNA-seq) sample, ribonucleic acid (RNA) extraction and library preparation were undertaken to remove globin messenger RNA (mRNA) and ribosomal RNA (RNAeasy Mini Kit [https://www.qiagen.com], Truseq stranded mRNA Library Prep Kit and Globin-zero Gold [https://www.illumina.com; RRID:SCR_010233]). RNA -seq was performed on an Illumina HiSeq platform (depth of 100 M paired-end reads and 2 × 150 bp read lengths; https://www.illumina.com). For whole genome sequencing, DNA was extracted using QIAamp DNA Mini Kit (https://www.qiagen.com; RRID:SCR_008539) and whole genome DNA sequencing (WGS) performed on an Illumina HiSeq platform at 2 × 150 bp paired-end reads to 50 × mean coverage across the human reference genome.

#### Bioinformatics analysis

The identification of small nucleotide and copy number variations, and mRNA expression was performed using the bcbio-nextgen (RRID:SCR_004316) platform 1.0.9 under default parameters [https://github.com/chapmanb/bcbio-nextgen]. In brief, sequencing reads were aligned in parallel to the human reference genome GRCh37 and mouse reference genome mm10 using BWA 0.7.17 (RRID:SCR_010910)^21^; only those reads that were better aligned to the human genome than to the mouse genome were retained using the PDX Disambiguate approach^22^. Subsequent to this alignment, small nucleotide variants were called using Vardict-Java 1.5.1 [10.1093/nar/gkw227] and artefactual mouse variants were removed using the PDX Blacklist method^23^. Copy number variations were called on the disambiguated alignments using CNVkit 0.9.2^24^. Annotations of small variants were performed using SNPeff 4.3 (RRID:SCR_005191)^25^ and Ensembl gene model 92 (RRID:SCR_002344); variants that occurred at ≥ 1% minor allele frequency in at least one ExAC normal population^26^ were omitted. Remaining variants with either a SNPeff predicted functional impact consequence of 'HIGH' or that were annotated as pathogenic in Clinvar 2017.0905 (RRID:SCR_006169)^27^ were considered to induce ‘loss-of-function’ (LoF) and were utilized for subsequent analysis. For RNA-seq data, mRNA expression was quantified as transcripts-per-million (TPM) using Salmon 0.9.1^28^ against a combined GRCh37/mm10 reference transcriptome index based on the human Ensembl gene model 92 at the gene level. Only those analyses on the human reference were retained and rescaled to a sum of 1 M TPM per sample. Quality control of both DNA and RNA analyses was undertaken using Samtools 1.7 (RRID:SCR_002105), FastQC 0.11.7 (RRID:SCR_014583)^29^ and Qualimap 2.2.2 (RRID:SCR_001209)^30^; all samples passed quality control.

### Statistical analyses

For the cytotoxicity data, the % values relative (%RV) to the vehicle controls were calculated for each dose level. The % inhibition values (%IV) were calculated by the formula: %IV = 100 − %RV. The calculated %IVs were used to calculate the EC50 values (µM) corresponding to 50% inhibition in ATP contents when compared to the vehicle controls for each experiment. The overall EC50s were the averages of the values obtained from each experiment (PRISM^®^ GraphPad™ Software, Boston, MA).

Tumor volume was used as the measure of efficacy for statistical analyses of in vivo xenograft studies. Tumor volume data from individual studies were log-transformed and statistically analyzed using repeated measures analysis of covariance (RM-ANCOVA) to determine significant treatment effects, followed by post hoc Tukey’s test for multiple pairwise comparisons (all α = 0.05) using R software. Day 0 data were used only as covariates in the analyses because tumor measurements on that day were recorded before the first treatments were administered.

For statistical analyses of bioinformatics data, the presence of LoF small nucleotide variations and copy number deletions were transformed into binary molecular readouts at the gene level. mRNA expression, TPM values, and integer allele copy number calls were taken directly as continuous molecular readouts at the gene level. In addition to single-gene molecular readouts, median mRNA expression values of molecular pathways were computed and investigated as additional, continuous molecular readouts.

For each gene and pathway assayed, statistical associations of molecular readouts with the continuous %∆T/∆C endpoint were computed across all samples. For binary molecular readouts, an unpaired non-parametric two-sided Wilcoxon rank-sum test with Pallant effect size was employed. For continuous readouts, a two-sided non-parametric Kendall rank correlation test with absolute Kendall's tau effect size was used. Only associations remaining after filtering with a *P*-value cutoff of 0.05 and effect size cutoff of 0.4 were considered for further analysis.

### Ethics

Procedures related to animal handling, care, and treatment in these studies were approved by the Institutional Animal Care and Use Committee (IACUC) of Crown Bioscience (Taicang, China), XenoSTART, Shanghai ChemPartner Co., Ltd, and EMD Serono Research and Development Institute. In vivo animal experiments at EPO-Gmbh were carried out in accordance with the United Kingdom Co-ordinating Committee of Cancer Research (UKCCCR) and its Guidelines for the Welfare of Animals in Neoplasia Research and the German Animal Protection Law, and approval was issued by local responsible authorities (LAGeSo Berlin, no. 0010/19). In vivo experiments were carried out in accordance with ARRIVE guidelines (Animal Research: Reporting of In Vivo Experiments).

When the PDX models at Crown Bioscience and XenoSTART were developed, they were established in immune-deficient mice with tumor tissue derived from patients with their informed consent and under approval of each respective Institutional Review Board. At the time of development, the use of human tumor tissue for the PDX models at EPO-GmbH was approved by the local Institutional Review Board of Charite University Medicine, Germany (EA4/019/12) and all patients had given written informed consent. All donations of tumor tissue by patients the time the PDX models were established were carried out in accordance with relevant guidelines and regulations.

## Results

### Potency and kinase selectivity

**Figure 1:**
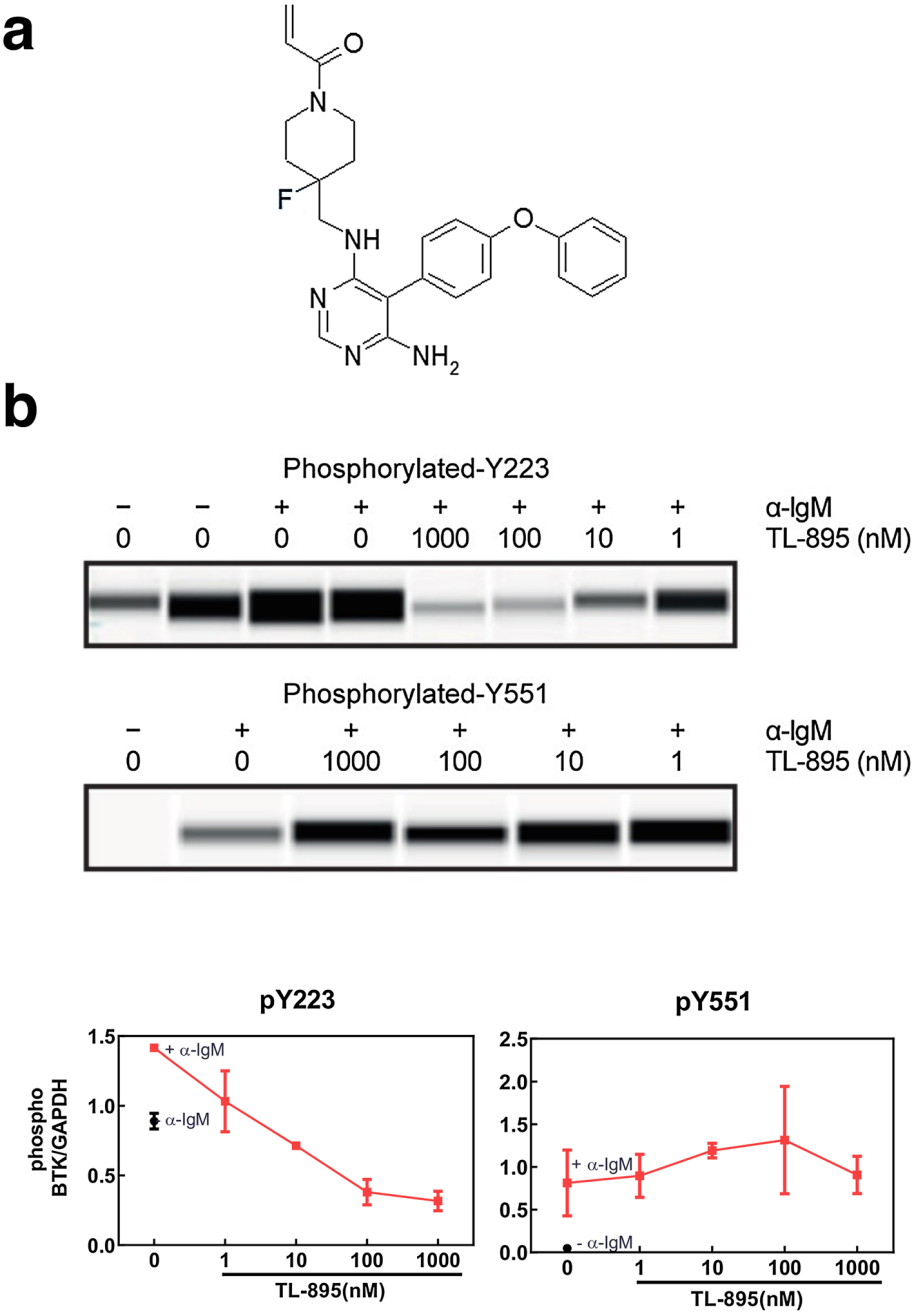
The chemical structure of TL-895 (**a**) and BTK autophosphorylation in Ramos cells following treatment with TL-895 after B-cell receptor stimulation (**b**). Top two panels of (**b**) show the ProteinSimple Wes results of BTK phosphorylation at Y223 and Y551 after pretreatment with increasing concentrations of TL-895 and in the presence of anti-IgM to stimulate BTK phosphorylation, and vehicle control in the presence and absence of anti-IgM. (The original Wes results from both runs are available in Supplementary Fig. S2.) Bottom panel shows the densities of the phosphorylated BTK bands normalized to the corresponding GAPDH bands and the percent inhibition for TL-895 calculated relative to the vehicle control cells activated with anti-IgM, shown as means of two gel runs ± standard deviation. *BTK* Bruton’s tyrosine kinase, *IgM* immunoglobulin M.

To measure the potency of TL-895 on the recombinant BTK protein, two methods were used. In an Off-Chip mobility biochemical assay, TL-895 inhibited recombinant BTK activity, with an average IC_50_ of 1.5 nM (n = 9, Supplementary Fig. S1). The Millipore Kinase Profiler screen kinome tree of TL-895 has been published^14^ and the structure of TL-895 is given in Fig. 1a. The Millipore Kinase Profiler screening panel, profiling across 270 protein kinases at 1 μM, showed an average IC_50_ for TL-895 on BTK was 18.5 nM (n = 2). Three additional kinases were also inhibited by TL-895 (Blk, BMX, and Txk) with IC_50_ values within a tenfold range of BTK (IC_50_ 77, 5, and 62 nM, respectively). A hallmark of irreversible covalent inhibitors is their time dependency. The differences in BTK IC_50_ for TL-895, as measured by the Off-Chip mobility assay and the radiolabel filter binding assays used in broad kinome profiling, may reflect differences in overall assay methodology.

### In vitro target inhibition of BTK

TL-895 inhibited BTK auto-phosphorylation at Y223 after BCR stimulation with anti-IgM in the human Burkitt’s lymphoma B-cell line Ramos in a concentration-dependent manner with an IC_50_ between 1 and 10 nM (Fig. 1b). Phosphorylation of Y551 on the activation loop was not inhibited, even at the highest tested concentration of TL-895 (1 µM) (Fig. 1b).

### Anti-proliferative effects in vitro and cytotoxicity

After specific, nanomolar inhibition of BTK was demonstrated in biochemical assays, the growth inhibitory effects of TL-895 on cells were examined in vitro. TL-895 inhibited growth of primary CLL blasts in vitro, similar to ibrutinib and acalabrutinib (Table 1), with an IC_50_ of approximately 0.2 µM for all three compounds. TL-895 had varying activity across DLBCL and MCL cell lines in vitro (Table 2). Four of six MCL cell lines reached growth inhibition levels of > 50% with TL-895. While GCB-DLBCL cell lines were not particularly sensitive to TL-895 (IC_50_s > 5 µM), a subset of ABC-DLBCL cell lines were sensitive (Table 2), with this sensitivity possibly linked to CD79/MyD88 mutation profiles. Both OCI-Ly3 and SU-DHL-2-epst, which harbor a MyD88 mutation in a CD79 wild-type background, were unresponsive to TL-895, whereas HBL-1 and TMD8, which have mutations in both CD79b and MyD88, were sensitive to TL-895.

**Table 1:** Mean growth inhibition (%) in primary CLL blasts.

Concentration (μM)	TL-895 (n = 21)	Ibrutinib (n = 21)	Acalabrutinib (n = 10)
25	90	97	80
5	72	76	64
1	61	63	56
0.2	53	57	47
0.04	50	47	40
0.008	43	43	34

*CLL* chronic lymphocytic leukemia.

**Table 2:** Anti-proliferative effects of TL-895 in MCL and DLBCL cell lines.

Cell line	GI_50_ (µM)	IC_50_ (µM)	Max response (GI %)
MCL			
GRANTA-519	NA	NA	50
Jeko-1	NA	NA	42
JVM-2	6.81	17.90	135
Maver-1	27.50	NA	53
Mino	5.58	14.00	88
REC-1	4.26	13.00	83
ABC-DLBCL			
HBL-1	0.56	10.30	89
OCI-Ly3	NA	NA	39
SU-DHL-2-epst	NA	NA	2
SU-DHL-8-epst	23.00	25.70	62
TMD8	0.01	0.01	181
U-2932	1.97	6.60	105
GCB-DLBCL			
DB	NA	NA	25
DOHH-2	9.39	11.40	130
HT	NA	NA	17
HU-DHL-1-epst	20.80	24.90	73
OCI-Ly18	18.60	22.30	68
OCI-Ly19	NA	NA	36
Pfeiffer	19.60	21.00	99
SU-DHL-4-epst	10.60	11.90	94
SU-DHL-5-epst	NA	NA	42
Toledo	3.84	5.18	195

*ABC-DLBCL* activated B-cell like diffuse large B-cell lymphoma, *IC*_*50*_ half maximal inhibitory concentration, *GCB-DLBCL* germinal B-cell like subtype-diffuse large B-cell lymphoma, *GI* growth inhibition, *GI*_*50*_ 50% growth inhibition, *MCL* mantle cell lymphoma, *NA* not available. NA, GI_50_ and IC_50_ values were not available as TL-895 did not reach growth inhibition levels > 50%. GI of 0% represents no growth inhibition, a GI of 100% represents complete growth inhibition (cytostasis) and a GI of 200% represents complete death (cytotoxicity) of all cells in the culture well.

Cytotoxicity assay EC50s of TL-895 in HepG2 cells (> 50 μM at 24 and 72 h) were 5–5000 fold greater than IC50s of the compound in sensitive MCL and DLBCL cell lines, and 32-fold greater in primary human hepatocytes (6.4 μM at 24 h) than in primary CLL blasts, showing a lack of cytotoxicity at doses that affect proliferation through BTK inhibition, with the caveat that the cytotoxicity in the hepatic cells may not be reflective of what occurs in leukemic cells.

### Effects on ADCC

**Figure 2:**
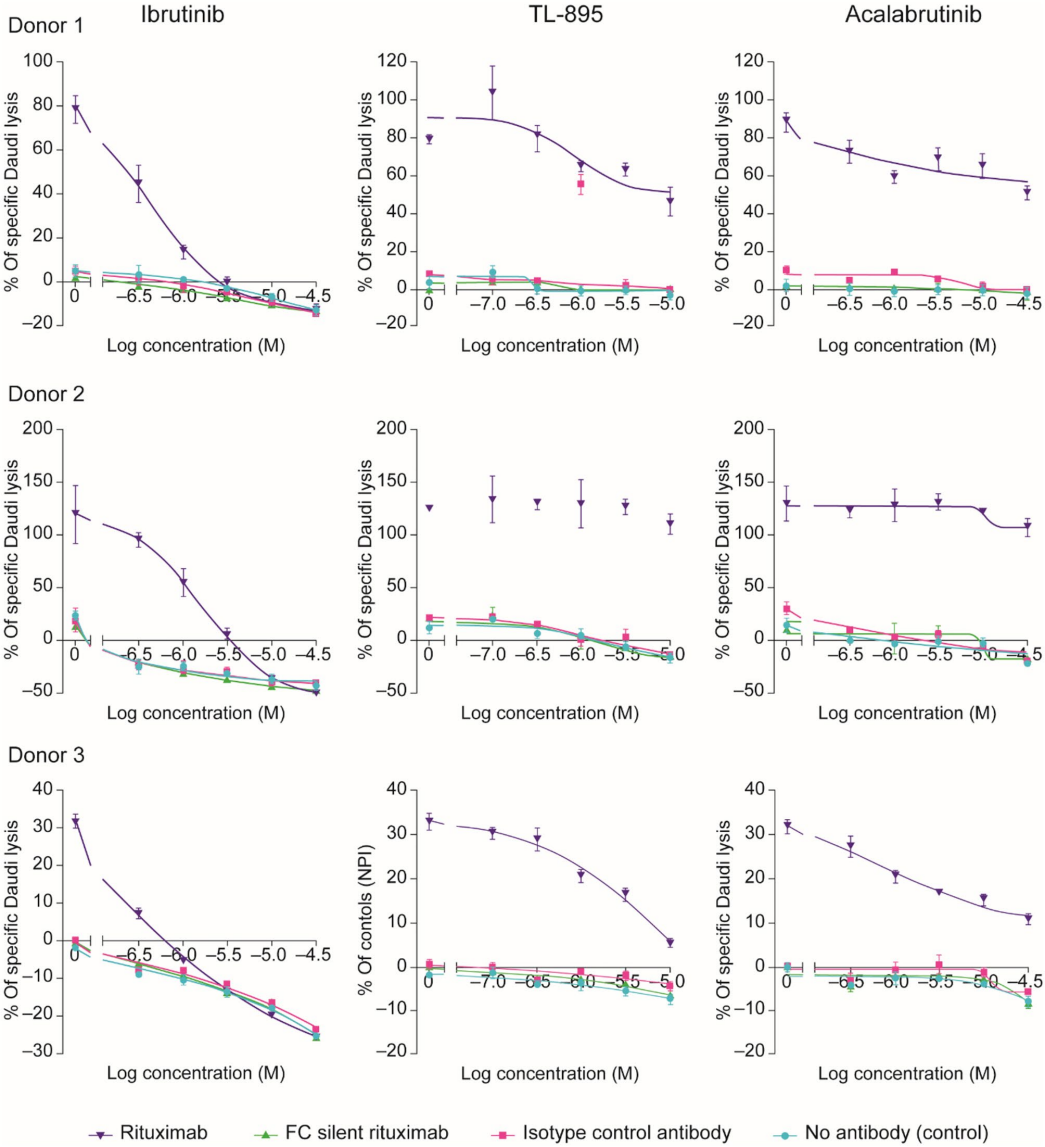
Effects of TL-895, ibrutinib and acalabrutinib on rituximab-induced ADCC in Daudi cells. Data are shown for three typical donors. *ADCC* antibody-dependent cell-mediated cytotoxicity.

ADCC was assessed by measuring the NK cell-driven lysis of Daudi cells. In this study, neither TL-895 (0.1–10 µM) nor acalabrutinib (0.3–30 µM) inhibited the ADCC mechanism of rituximab at the lower, more clinically relevant, concentrations (based on doses investigated in a phase 1 study^20^), whereas ADCC was inhibited by ibrutinib (0.3–30 µM) in a concentration-dependent manner (Fig. 2).

**Figure 3:**
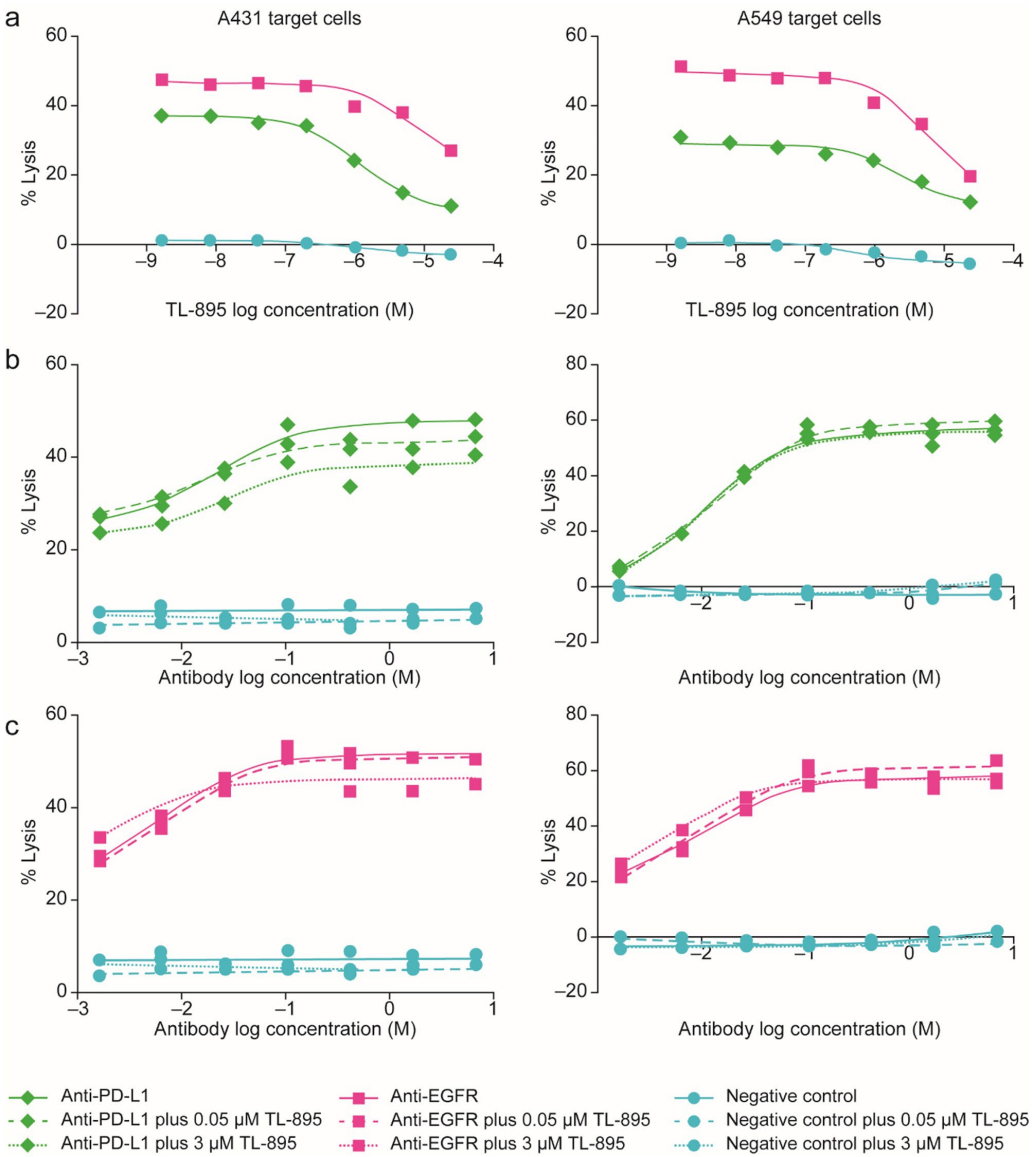
Impact of TL-895 on the ADCC of anti-PD-L1 and anti-EGFR (C225) antibodies. Conducted in two target cell lines (A431 and A549), where panel (**a**) shows single concentrations (20 ng/mL) of anti-PD-L1 and anti-EGFR (C225) antibodies with a range of concentrations of TL-895; panels (**b**) and (**c**) show 0.05 or 3 µM TL-895 with a range of concentrations of anti-PD-L1 antibody and anti-EGFR antibody, respectively. *ADCC* antibody-dependent cell-mediated cytotoxicity, *EGFR* epidermal growth factor receptor, *PD-L1* programmed death-ligand 1.

In the second study, TL-895 did not affect the concentration–response or maximum cell lysis when combined with anti-PD-L1 or anti-EGFR antibodies, except at the highest concentrations tested (Fig. 3), which represent supra-therapeutic concentrations.

### Cell line-derived mouse xenograft models of MCL

**Figure 4:**
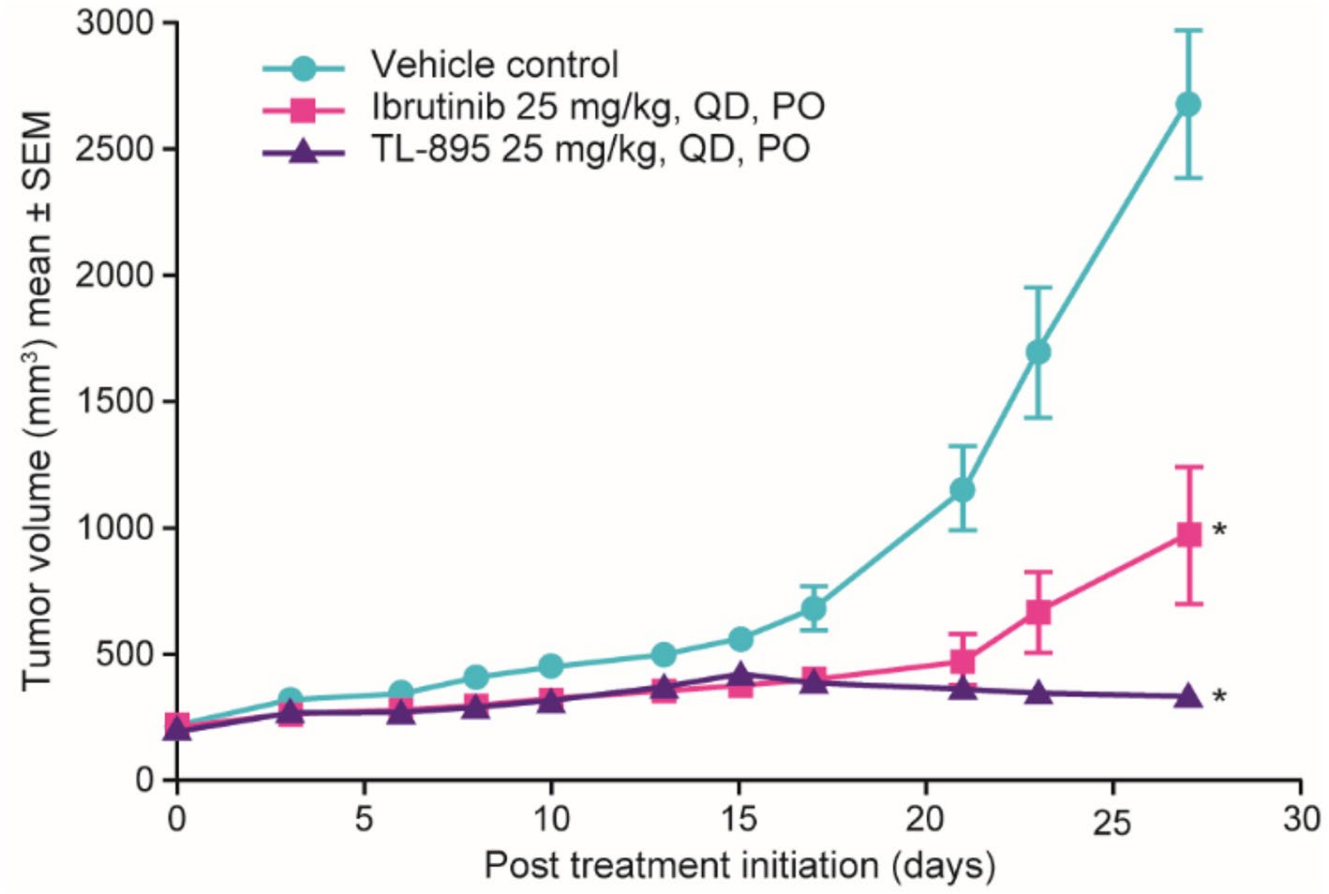
Volume of Mino tumors in mice treated with vehicle, TL-895, or ibrutinib. Tumor-bearing mice were dosed PO, QD for 27 days. Significant treatment effects were observed for TL-895 and ibrutinib versus vehicle (*P < 0.05), but not compared with each other (*P* > 0.05). *PO* orally, *QD* once daily.

Based on the observed sensitivity of MCL cell lines in vitro*,* TL-895 was evaluated for its therapeutic benefit in four mouse models of MCL. TL-895 significantly inhibited tumor growth in the Mino cell line-derived xenograft model of MCL compared with vehicle (*P* < 0.001) (Fig. 4). No significant effects due to treatment by TL-895 versus ibrutinib were noted (*P* > 0.05). Neither TL-895 nor ibrutinib had any significant effects on tumor growth in the Jeko-1, Maver-1, and Granta519 models (all *P* > 0.05 vs vehicle) (Supplementary Fig. S3).

### PDX models of DLBCL

Based on molecular analyses, the 21 DLBDL PDX models were classified as ABC (*n* = 9) or GCB (*n* = 11) subtypes, with one sample unclassified (Table 3); four models were found to harbor a MyD88 mutation and none of the models had a CD79 mutation. Inhibition of tumor growth by BTK inhibition was most effective in DLBCL PDX models of the ABC subtype (Table 3). Treatment with TL-895 and acalabrutinib significantly inhibited tumor growth compared to vehicle (*P* < 0.05) in four different ABC models and in one GCB-DLBCL PDX model, whereas tumor growth was significantly inhibited by ibrutinib compared with vehicle in only one ABC (*P* < 0.05) and one GCB-DLBCL model (both of which were also inhibited by TL-895 and acalabrutinib; Table 3).

**Table 3:** Effects of TL-895, ibrutinib and acalabrutinib on growth of DLBCL PDX tumors.

Model ID/subtype	Treatment duration (days)	TL-895 (%ΔT/ΔC*)	Ibrutinib (%ΔT/ΔC*)	Acalabrutinib (%ΔT/ΔC*)
LY0257/ABC^MyD88^	17	45^V,I,A^	88^M^	54^M^
LY13005/ABC	14	43^V,I^	71^M^	64^V^
LY2298/ABC^MyD88^	16	38	69^A^	27^V,I^
LY2264/ABC^MyD88^	25	37^V,I^	74^M^	48^V^
LY2345/ABC	17	120	118	108
ST949B/ABC	9	39	96	103
LY3604/ABC	17	32^V^	38^V^	36^V^
ST118B/ABC^MyD88^	19	68	90	104
LY2318/ABC	14	104	86	102
LY11212/GCB	13	115	171	126
ST2078/GCB	20	114	75^A^	115^I^
ST2963/GCB	13	106	81	116
ST2902/GCB	22	104	92	85
ST1735B/GCB	29	101	75	80
ST23497/GCB	17	101	95	100
ST2761B/GCB	24	90	71	94
LY12638/GCB	25	84	121	262
ST949/GCB	14	83	86	72
ST359/GCB	11	71	62	54
ST1361/GCB	11	60^V^	65^V^	74^V^
LY2214/Unc	17	93	77	63

*%∆T/∆C* tumor growth inhibition expressed as the mean percentage treatment (T)/control (C) ratio, *DLBCL* diffuse large B-cell lymphoma, *MyD88* models with mutations in *MyD88*PDX, *PDX* patient-derived xenograft, *Unc* unclassified. No models were found to have CD79 mutations. *Significantly different (*P* < 0.05) versus: ^A^, acalabrutinib; ^I^, ibrutinib; ^M^, TL-895; ^V^, vehicle.

There was no mean tumor regression for any of the treatments in any of the models (Supplementary Fig. S4), and tumor regression in individual mice was relatively rare. Using statistical significance (*P* < 0.05) of treatments versus vehicle as a threshold for tumor response in these DLBCL models (Table 3), the tumor response rate was 24% (*n* = 5/21 models) for both TL-895 and acalabrutinib, and 10% (*n* = 2/21 models) for ibrutinib.

### Biomarker analyses in PDX DLBCL models

**Figure 5:**
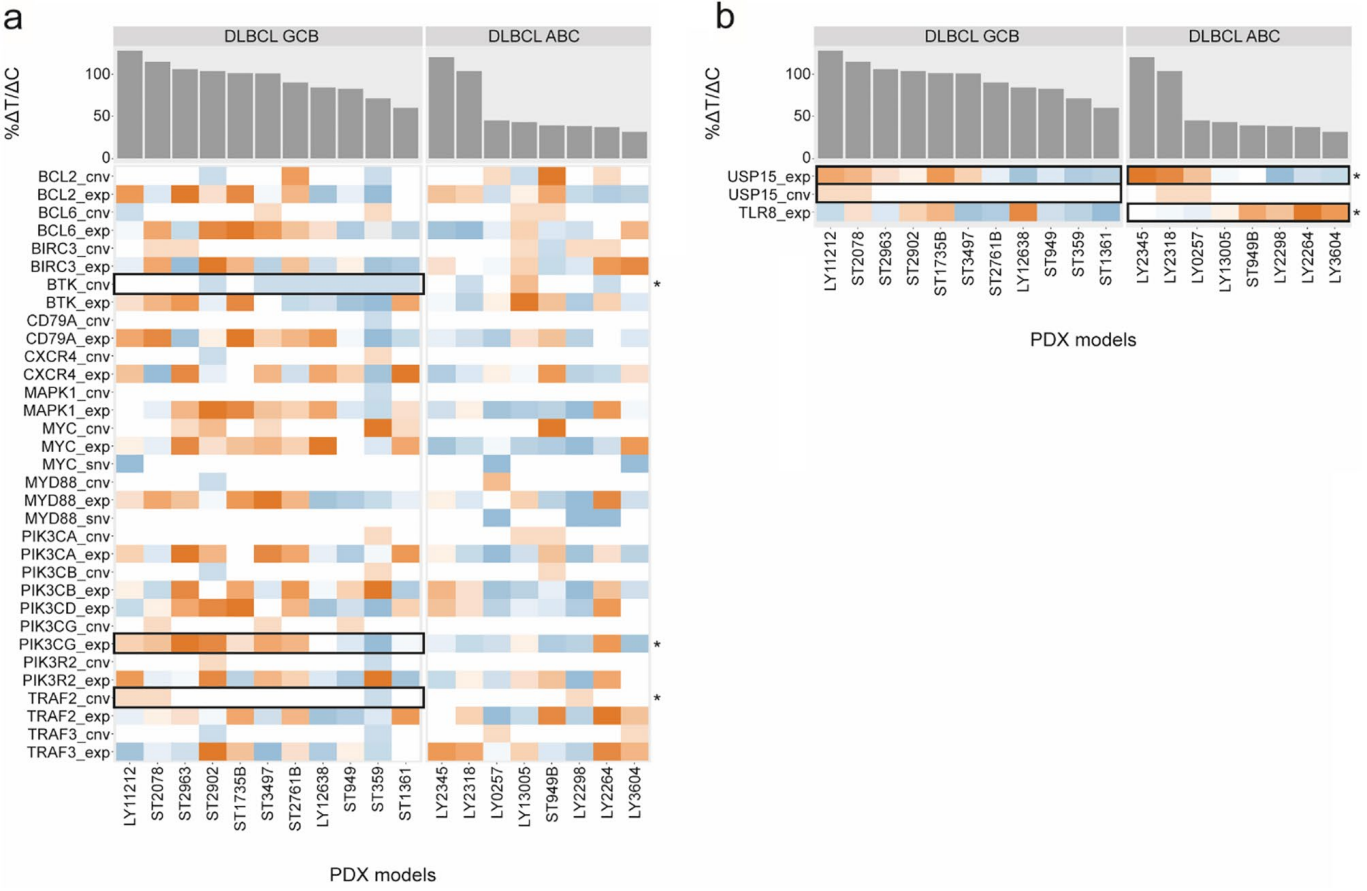
Association of tumor response with biomarker status in PDX models of DLBCL. Data in panels represent (**a**) hypothesis-driven biomarker analysis and (**b**) data-driven biomarker analysis. Bar charts show tumor response (%∆T/∆C) following TL-895 treatment by DLBCL subtype (GCB and ABC) with heatmaps of the corresponding molecular readouts of rank-normalized mRNA gene expression (exp), gene copy number (cnv) and gene mutation (snv) for each model. Colors of the heatmap represent the row-wise normalized value of the readout. For exp and cnv, red colors denote high values and blue colors denote low values. For single nucleotide variants, blue indicates a mutation while white indicates the wild type. Genes of interest are denoted by their gene symbols. Black rectangles highlight associations that passed statistical association tests (*P* < 0.05). *DLBCL* diffuse large B-cell lymphoma, *PDX* patient-derived xenograft.

Using efficacy data from the PDX DLBCL models, RNA sequencing (RNAseq) gene expression analyse and mutation and CNV profiles of each PDX model were performed to identify molecular correlates of sensitivity to TL-895 treatment (Fig. 5).

A hypothesis-driven biomarker analysis evaluated BTK-related genes. There was a statistically significant association (*P* < 0.05) for PIK3CG in the GCB subtype, where models with lower expression of PIK3CG showed a higher sensitivity to TL-895. This association was not observed in the ABC subtype models. Likewise, a significant association between sensitivity to TL-895 and BTK and TRAF2 copy number status was only observed in the GCB subtype, where GCB-DLBCL PDX models with copy number loss of BTK and TRAF2 showed most sensitivity to TL-895 (Fig. 5a).

A broader, data-driven biomarker analysis was performed, where we evaluated NFκB-, immune-, and cancer-related genes, as well as the interaction partners of these genes and of the BTK-related genes. In addition, we evaluated associations at the pathway-level, where definitions of pathways were obtained from MsigDB, Pathway Commons, CORUM protein complexes, and PhosphoSitePlus. Significant correlations were observed for TLR8 in the ABC subtype models, where models with higher expression showed a greater sensitivity to TL-895, and USP15 in both ABC and GCB subtype models, where models with lower expression showed a greater sensitivity to TL-895 (all *P* < 0.05, Fig. 5b).

## Discussion

TL-895 is a second-generation BTK inhibitor that demonstrates high potency and good selectivity. With fewer off-target activities, TL-895 may provide the efficacy associated with the first-generation BTK inhibitor, ibrutinib, but with a reduced likelihood of adverse events for patients. In the current report, the potency and preclinical efficacy of TL-895 were characterized through a series of in vitro and in vivo studies and compared with those of ibrutinib and another second-generation BTK inhibitor, acalabrutinib. These studies confirmed the refined selectivity profile of TL-895, which inhibited only three off-target kinases (Blk, BMX, and Txk) (based on IC_50_ values within a tenfold range of that for BTK).

BTK inhibition by TL-895 was seen with a concentration-dependent decrease in BTK auto-phosphorylation in the Ramos cell line (human Burkitt’s B-cell lymphoma) in one study, with the caveat that these were results from a single experiment. If the data are representative of the mechanism in patient leukemic cells, the inhibition of BTK phosphorylation at the Y223, but not Y551 site, suggests that the molecular mechanism of TL-895 involves preventing the phosphorylation of the BTK Y223 moiety through direct inhibition of the kinase domain. Phosphorylation of BTK occurs sequentially, with Y551 phosphorylated first after BCR activation, which promotes its catalytic activity and subsequently results in the autophosphorylation of Y223^31^. Phosphorylation at both sites is believed to be required for kinase activity of the protein; TL-895 may bind to the BTK active site in a way that does not prevent the conformational change triggered by Y551 phosphorylation but does prevent activation of the enzyme that leads to autophosphorylation of BTK at Y223.

After establishing the selectivity of TL-895, its activity in vitro and in vivo was investigated. The action of TL-895 on BTK in vitro translated into inhibition of the growth of primary CLL blasts, MCL cells, and a subset of B-cell malignancy cell lines. TL-895 had a similar inhibitory effect to that of ibrutinib and acalabrutinib in CLL cell lines and most MCL cell lines were moderately sensitive to TL-895. Although TL-895 was not tested in vivo in CLL models, TL-895 treatment in vivo inhibited tumor growth in one of four MCL models, which was similar to the effects seen with ibrutinib. As such, the improved off-target profile of TL-895 maintained its in vivo efficacy relative to that of ibrutinib.

Consistent with previous reports for other BTK inhibitors^6^, TL-895 had greater activity in the ABC subset of DLBCL models, and limited inhibition of GCB models, both in vitro and in vivo. Although the in vitro ABC-DLBCL cell line panel used in our studies was relatively small, there was a potential trend for sensitivity based on CD79/MyD88 mutation profiles, as seen previously with ibrutinib^6^. Unfortunately, this trend could not be evaluated in the PDX models, as none of the models had CD79 mutations. However, tumor growth in PDX DLBCL models was inhibited to different extents with TL-895, ibrutinib, and acalabrutinib, although tumor regression was not observed. This lack of regression is in contrast to clinical observations where ibrutinib and acalabrutinib monotherapy have resulted in partial and complete responses in patients with DLBCL^6,32^; there is some preliminary evidence with TL-895 (1 complete response^33^). The tumor response exhibited in these PDX DLBCL models suggests that they have relatively lower sensitivity to BTK inhibition with TL-895, ibrutinib, and acalabrutinib, when compared with clinical observations. Therefore, the translatability of the outcomes from these models was based on statistically significant treatment effects in comparison to vehicle.

The tumor response rate for ibrutinib in the PDX models in our studies was 10%, which compares with reported rates of 37% in patients with ABC-DLBCL and 5% in those with GCB-DLBLC^6^. The calculated tumor response rates for TL-895 and acalabrutinib (both 24%) suggest that these two second-generation BTK inhibitors have superior efficacy in these DLBCL PDX models; for acalabrutinib, the rate is consistent with clinical observations in patients with either ABC- or GCB-DLBCL (combined overall response rate [ORR] of 24%^32^). In contrast, the relatively low tumor response of 10% with ibrutinib in these DLBCL PDX models is not consistent with clinical observations of ibrutinib in patients with wild type CD79B ABC-DLBCL (ORR 31%^6^), or when combined with R-ICE (rituximab, ifosfamide, carboplatin, and etoposide) in patients with GCB-DLBCL (ORR 90%^34^). The antitumor activity of TL-895 is similar to that of acalabrutinib and twice that of ibrutinib, indicating the promise of TL-895 in DLBCL.

Ibrutinib is known to have inhibitory effects on ADCC, which has been attributed to its off-target inhibition of ITK^35–37^. Therefore, potential inhibition of effector cell responses to therapeutic antibodies that induce ADCC, such as rituximab, has been a concern. TL-895 was designed to have a refined pharmacological profile that would maintain the effectiveness against the target but would not inhibit effector cell functions such as ADCC. At clinically relevant concentrations, ibrutinib inhibited ADCC induced by rituximab, whereas TL-895 and acalabrutinib did not. The lack of any inhibition of ADCC by TL-895 was further substantiated using anti-EGFR and anti-PD-L1 antibodies. It is possible, therefore, that TL-895 may be a better option than ibrutinib for clinical use in combination with ADCC-competent therapeutic antibodies.

Taken together, the data presented here confirm the high selectivity of the second generation BTK inhibitor, TL-895, and demonstrate that its potent antitumor effects in B-cell malignancies are BTK target related, and not compromised by its refined selectivity profile. Second generation BTK inhibitors have been designed to increase selectivity toward BTK while limiting the inhibition of similar kinases, as a means to circumvent some of the off-target toxicities identified with ibrutinib, such as atrial fibrillation^38^. The translation of the preclinical selectivity profile of TL-895 into potential clinical benefits has been described in the first-in-human phase 1/2 study of TL-895 conducted in patients with B-cell malignancies^20,33^. Preliminary evidence from that clinical study supports potent antitumor activity and a well-tolerated safety profile. In that study, no dose-limiting toxicities were reported with TL-895 at doses up to 900 mg/day and the maximum tolerated dose was not reached. Most of the reported adverse events were classed as mild-to-moderate, and three patients (17%) experienced treatment-related grade 3–4 adverse events^33^. Clinical benefit was observed even at the lowest dose of TL-895, and across the tumor types. Half of patients had a best response of PR or higher, which is in the general range of other BTK inhibitors as monotherapy^3,4,39,40^.

## Conclusions

Collectively, these findings demonstrate the potency of TL-895 for BTK and its efficacy in models of B-cell lymphoma despite its refined selectivity.

### Supplementary Information

Supplementary Information.

## Data Availability

The datasets generated and analyzed during the current studies are not publicly available due to their large size, but these are available from the corresponding author (SG) on reasonable request.

## Abbreviations

AAALAC: Association for Assessment and Accreditation of Laboratory Animal Care
ABC: Activated B-cell like
GCB: Germinal B-cell like subtype
ADCC: Antibody-dependent cell-mediated cytotoxicity
ATP: Adenosine triphosphate
AUC: Area under the curve
BTK: Bruton’s tyrosine kinase
CLL: Chronic lymphocytic leukemia
DLBCL: Diffuse large B-cell lymphoma
EDTA: Ethylenediamine tetra-acetic acid
EGFR: Epidermal growth factor receptor
IACUC: Institutional Animal Care and Use Committee
IC_50_: Half maximal inhibitory concentration
IgM: Immunoglobulin M
ITK: Interleukin-2 inducible T-cell kinase
MCL: Mantle-cell lymphoma
PD-L1: Programmed death-ligand 1
PDX: Patient-derived xenograft
PO: Orally
QD: Once daily
