# Supplementary material for: Preclinical evidence for the effective use of TL-895, a highly selective and potent second-generation BTK inhibitor, for the treatment of B-cell malignancies
Source: Sci Rep. 2023 Nov 21;13:20412. doi: 10.1038/s41598-023-47735-z (PMC10663516; doi:10.1038/s41598-023-47735-z)
Supplement: Supplementary file 1 — Supplementary Information. [file 41598_2023_47735_MOESM1_ESM.pdf]

Supplementary material

Preclinical evidence for the effective use of TL-895, a highly selective and potent second-generation BTK inhibitor, for the treatment of B-cell malignancies

Samantha M. Goodstal, Jing Lin, Timothy Crandall, Lindsey Crowley, Andrew T. Bender, Albertina Pereira, Maria Soloviev, John S. Wesolowski, Riham Iadevaia,
Sven-Eric Schelhorn, Edith Ross, Federica Morandi, Jianguo Ma, Anderson Clark

Supplementary Methods

Kinase assay buffer components

Dilution buffer

4 mM dithiothreitol (Sigma, D0632)

10 mM $MgCl_2$ (Sigma, M1028)

100 mM 4-(2-hydroxyethyl)-1-piperazineethanesulfonic acid (HEPES) pH 7.5 (Calbiochem, 391338)

0.015% Brij-35 (Sigma, B4184)

Stop buffer

100 mM HEPES pH 7.5

0.015% Brij-35

10 mM EDTA

0.227% Coating Reagent #3 (Caliper Life Sciences)

5% DMSO

Deionized water

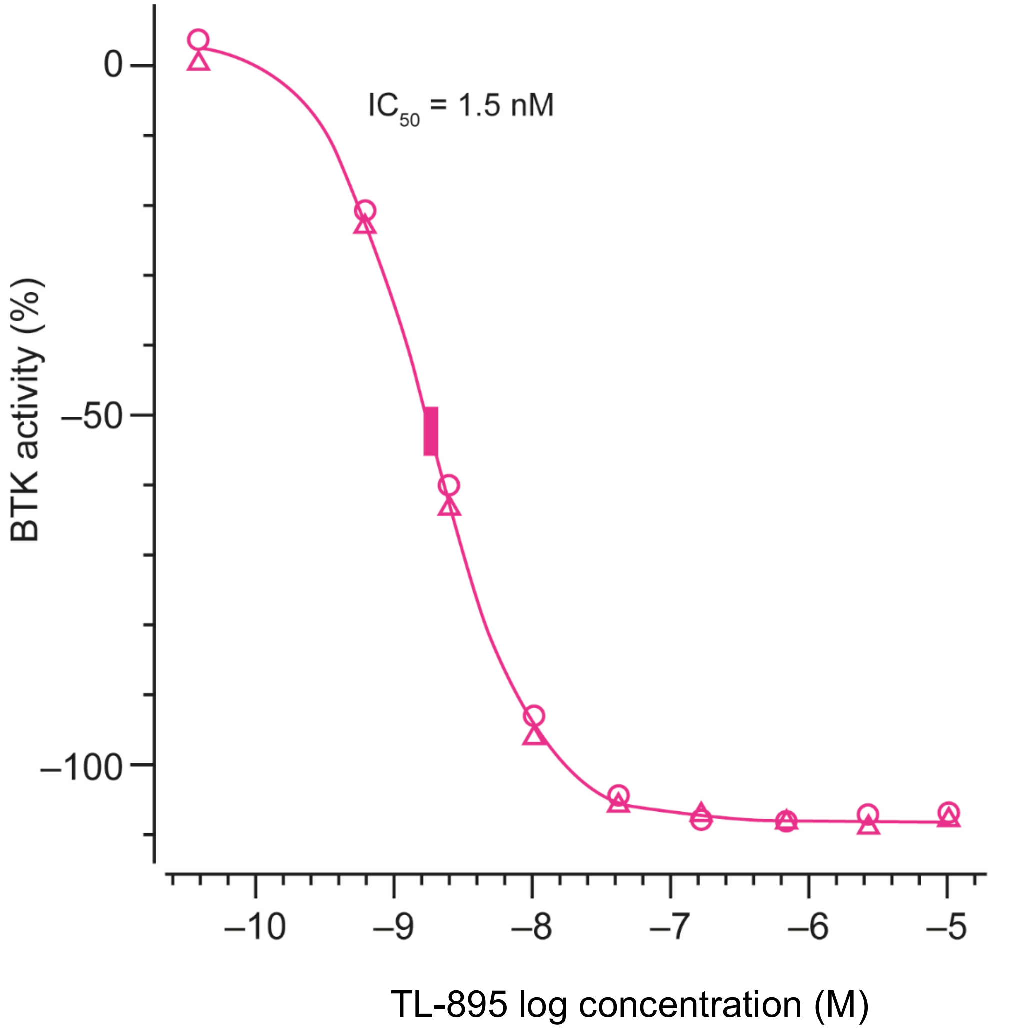

Supplementary Figure S1. Inhibition of recombinant human full-length BTK activity by TL-895 and resulting IC_{50}. BTK, Bruton's tyrosine kinase

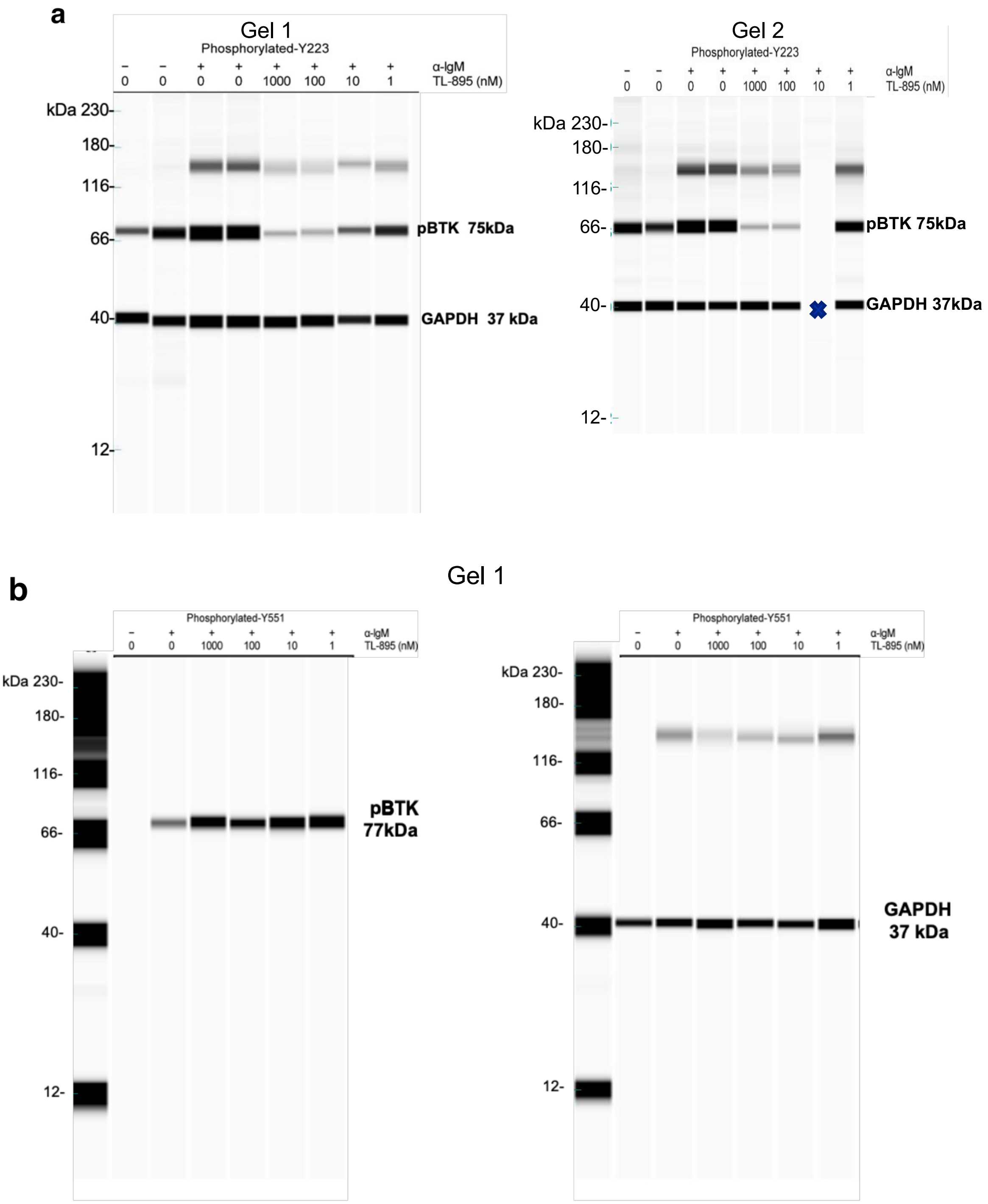
a
Gel 1
Phosphorylated-Y223
α-IgM
TL-895 (nM)
− − + + + + + +
0 0 0 0 1000 100 10 1
kDa 230-
180-
116-
66-
40-
12-
pBTK 75kDa
GAPDH 37 kDa
Gel 2
Phosphorylated-Y223
α-IgM
TL-895 (nM)
− − + + + + + +
0 0 0 0 1000 100 10 1
kDa 230-
180-
116-
66-
40-
12-
pBTK 75kDa
GAPDH 37kDa
b
Gel 1
Phosphorylated-Y551
α-IgM
TL-895 (nM)
− + + + + +
0 0 1000 100 10 1
kDa 230-
180-
116-
66-
40-
12-
pBTK
77kDa
Phosphorylated-Y551
α-IgM
TL-895 (nM)
− + + + + +
0 0 1000 100 10 1
kDa 230-
180-
116-
66-
40-
12-
GAPDH
37 kDa

Gel 2

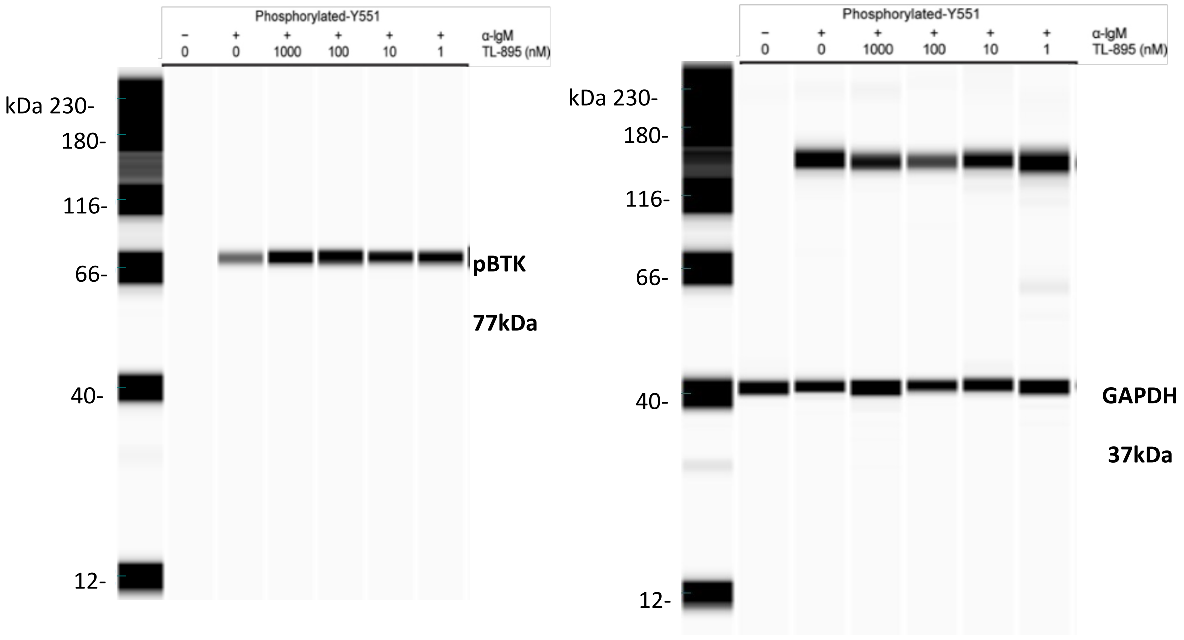

Supplementary Figure S2. BTK autophosphorylation in Ramos cells following treatment with TL-895 after B-cell receptor stimulation. Full length ProteinSimple Wes results from two gels shown of BTK phosphorylation at Y223 (a) and Y551 (b) after pretreatment with increasing concentrations of TL-895 and in the presence of anti-IgM to stimulate BTK phosphorylation, or vehicle control in the presence and absence of anti-IgM. In (a), pBTK and GAPDH were probed together whereas in (b), they were probed separately because different secondary antibodies were used. The larger molecular weight bands in (a) and (b) in the right panels are non-specific binding of the GAPDH antibody. Molecular weight markers are in the first lanes on the left. BTK = Bruton's tyrosine kinase; IgM = immunoglobulin M.

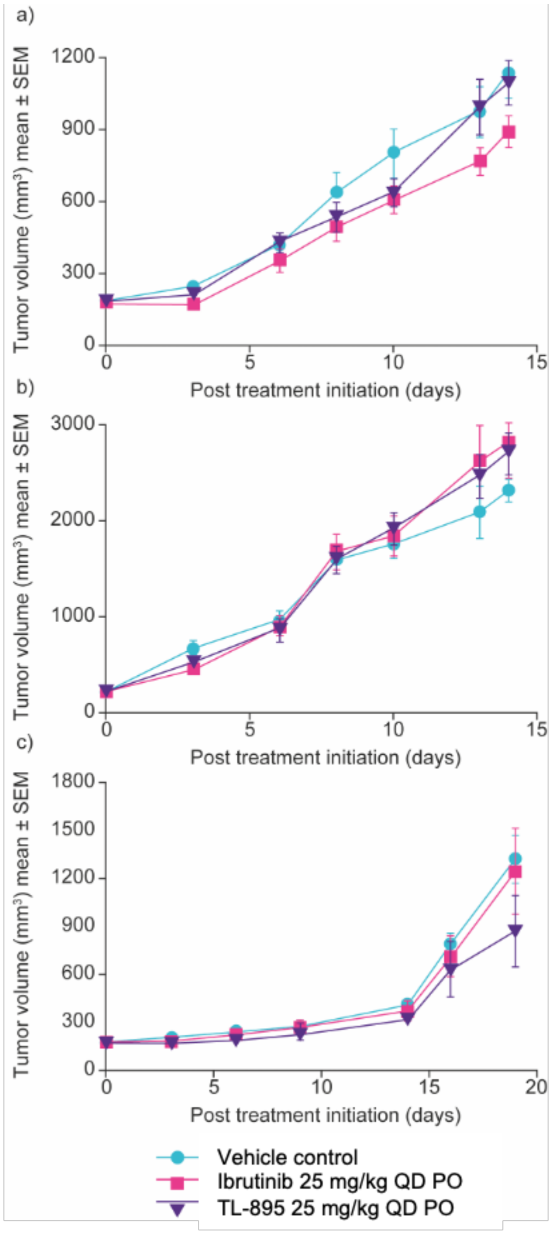

Supplementary Figure S3. Mantel cell lymphoma (MCL) xenograft tumors during treatment with ibrutinib or TL-895. Data represent tumor volume in mouse models of three MCL xenograft tumors; (a) Maver-1, (b) Granta519, and (c) Jeko-1. Animals were treated daily (QD) per os (PO). There were no sigificant treatment effects in any model ($p > 0.05$). Day 0 is 21 days after cell implantation.

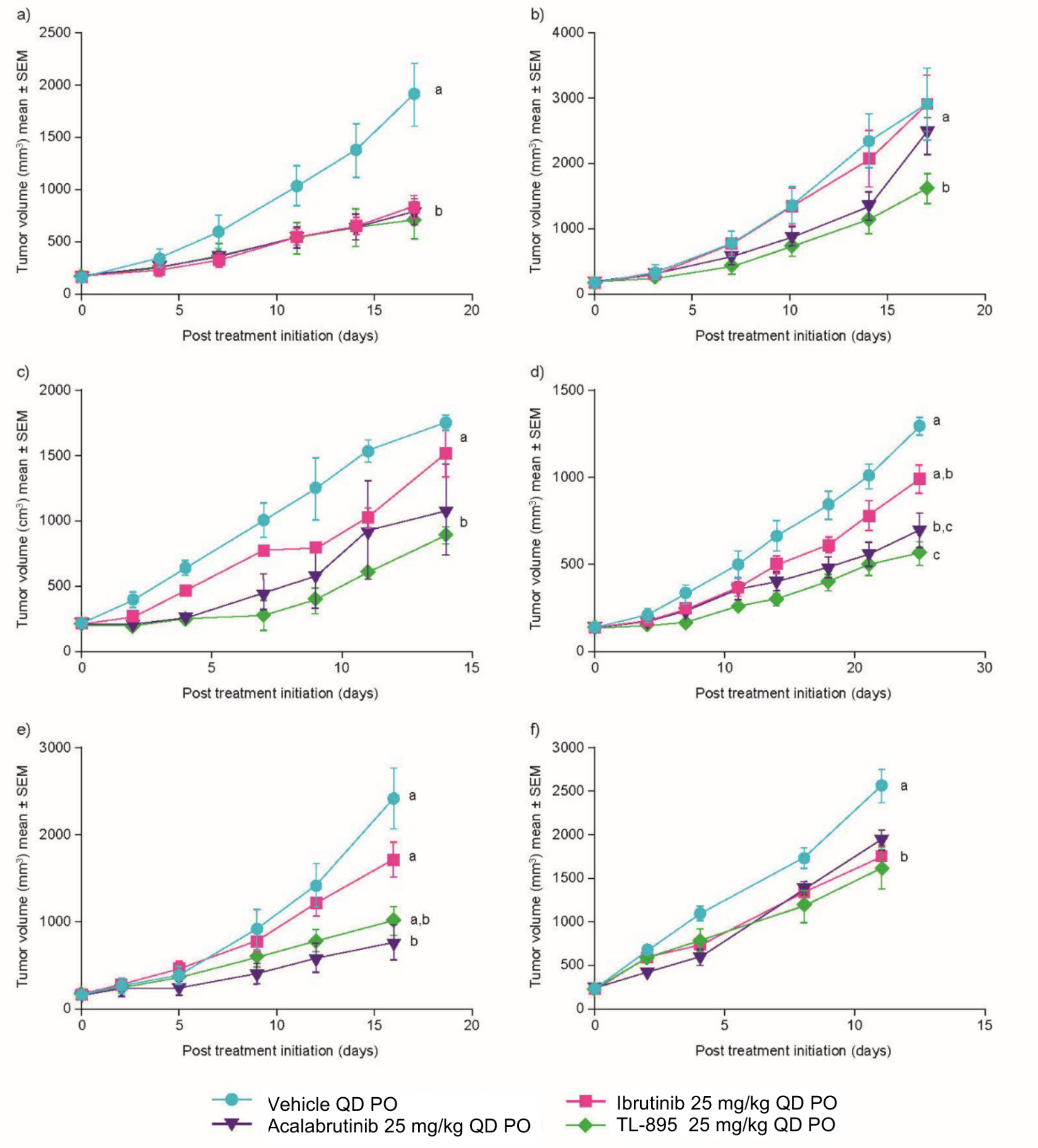

Supplementary Figure S4. Tumor volume in diffuse large B-cell lymphoma (DLBCL) patient-derived xenograft (PDX) models treated with TL-895, ibrutinib, or acalabrutinib. Data from PDX models of ABC DLBCL: (a) LY3604; (b) LY0257; (c) LY13005; (d) LY2264; (e) LY2298; and GCB DLBCL (f) ST1361. [a,b,c] Means with the same superscripts are not different ($p > 0.05$)
